# Differences in temperature responses among phenological processes in diverse Ethiopian sorghum germplasm can affect their specific adaptation to environmental conditions

**DOI:** 10.1093/aob/mcad011

**Published:** 2023-01-20

**Authors:** Alemu Tirfessa, Greg McLean, Peter Baker, Miranda Mortlock, Graeme Hammer, Erik van Oosterom

**Affiliations:** The University of Queensland, Queensland Alliance for Agriculture and Food Innovation, St Lucia, QLD 4072, Australia; Ethiopian Institute of Agricultural Research (EIAR), Melkassa Agricultural Research Center, PO Box 436, Adama, Ethiopia; Agri-Science Queensland, Department of Agriculture and Fisheries, Toowoomba, QLD 4350, Australia; The University of Queensland, School of Public Health, St Lucia, QLD 4072, Australia; The University of Queensland, Queensland Alliance for Agriculture and Food Innovation, St Lucia, QLD 4072, Australia; Queensland University of Technology, Brisbane City, QLD 4000, Australia; The University of Queensland, Queensland Alliance for Agriculture and Food Innovation, St Lucia, QLD 4072, Australia; The University of Queensland, Queensland Alliance for Agriculture and Food Innovation, St Lucia, QLD 4072, Australia

**Keywords:** Agroecological adaptation, base temperature, development rate, leaf appearance rate, leaf number, sorghum race, temperature response

## Abstract

**Background and Aims:**

Main shoot total leaf number (TLN) is a key determinant of plant leaf area and crop adaptation. Environmental factors other than photoperiod can affect TLN in sorghum, implying that leaf appearance rate (LAR) and development rate can differ in response to temperature. The objectives of this study were to determine (1) if temperature effects on TLN can be explained as a consequence of differences in temperature responses across phenological processes and (2) if genotypic differences in these responses can be linked to agroecological adaptation.

**Methods:**

Nineteen sorghum genotypes were sown on 12 dates at two locations in Ethiopia with contrasting altitude, creating temperature differences independent of photoperiod. TLN and temperature were recorded in all experiments and LAR for six sowing dates.

**Key Results:**

Eleven of the genotypes showed a temperature effect on TLN, which was associated with a significantly higher base temperature (*T*_base_) for LAR than for pre-anthesis development rate (DR). In contrast, genotypes with no effect of temperature on TLN had similar *T*_base_ for LAR and DR. Across genotypes, *T*_base_ for LAR and DR were highly correlated, but genotypes with low *T*_base_ had the greatest difference in *T*_base_ between the two processes. Genotypic differences were associated with racial grouping.

**Conclusions:**

Genotypic and racial differences in responses of phenological processes to temperature, in particular in *T*_base_, can affect specific adaptation to agroecological zones, as these differences can affect TLN in response to temperature and hence canopy size and the duration of the pre-anthesis period. These can both affect the amount of water used and radiation intercepted pre-anthesis. A multi-disciplinary approach is required to identify genotype × environment × management combinations that can best capture the ensuing specific adaptation.

## INTRODUCTION

Phenology is an important aspect of crop adaptation, as the timing of anthesis can play a significant role in minimizing the adverse effects of end-of-season drought stress on crop yields (Messina *et al.*, 2011). This is particularly the case for sorghum (*Sorghum bicolor*), which is usually grown under rainfed conditions in semi-arid tropical and sub-tropical environments, where the timing and severity of drought stress can be highly variable in both space and time (Kholová *et al.*, 2013; Hammer *et al.*, 2014). In sorghum, timing of anthesis is a function of the total leaf number (TLN) produced by the main shoot and the rate at which leaf ligules appear to mark full expansion of a leaf (leaf appearance rate, LAR, leaves d^−−1^ °C^−1^), which in turn depends on temperature and photoperiod.

TLN in sorghum arises from the four leaf initials present in the seed (Paulson, 1969) and the number of leaf primordia initiated at the apical meristem prior to panicle initiation (PI), when the apical meristem transitions from initiating leaf primordia to initiating reproductive organs. The number of leaf primordia initiated is thus the product of the leaf initiation rate (LIR, leaves d^−1^ °C^−1^) and the duration of the period between plant emergence and PI (°Cd), which in turn depends on the pre-anthesis phenological development rate (DR). LIR, LAR and DR (and thus PI) each depend on temperature, and the response is such that rates will be zero when temperature is below a base temperature (*T*_base_) or above a maximum temperature (*T*_max_) and rates will be highest at the optimum temperature (*T*_opt_) (Ong and Monteith, 1985; Hammer *et al.*, 2010). In general, DR, LIR and LAR are assumed to have similar cardinal temperatures (*T*_base_, *T*_opt_, *T*_max_) (Parent and Tardieu, 2012). Such a common response to temperature means that any change in DR (and hence timing of PI) in response to changes in temperature will be compensated for by a change in LIR, such that the total number of leaves initiated (and hence TLN) will be independent of temperature. This provides a simple model to predict the timing of anthesis of sorghum, based on estimated TLN and the response of LAR to temperature (Hammer *et al.*, 2010).

As sorghum is a short-day crop, the duration (°Cd) from emergence to PI will be extended for photoperiod-sensitive genotypes if the photoperiod prior to PI exceeds a threshold (Major *et al.*, 1990; Ravi Kumar *et al.*, 2009). This delayed occurrence of PI will extend the duration of leaf initiation, resulting in increased TLN (Muchow and Carberry, 1990; Ravi Kumar *et al.*, 2009). Although the assumption of common cardinal temperatures for DR, LIR and LAR implies that TLN will only be affected by photoperiod, there are reports for cereals of temperature effects on TLN that are independent of daylength. Increased TLN in response to higher temperature has been reported for sorghum (Caddel and Weibel, 1971; Gerik and Miller, 1984; Craufurd *et al.*, 1998; van Oosterom *et al.*, 2011) and maize (Cooper and Law, 1978; Warrington and Kanemasu, 1983), although there is some evidence that high temperature only increases TLN above a certain temperature threshold (Craufurd *et al.*, 1998). Observations from controlled environments, where temperature during the transition from dark to light affects the timing of PI (Morgan *et al.*, 1987; Ellis *et al.*, 1997), would imply a possible role of night temperature. Temperature effects on TLN that are independent of photoperiod could indicate a difference between LIR and pre-anthesis DR in their response to temperature, such that increased temperature will have different effects on these two processes, potentially increasing the number of leaves that are initiated prior to PI. Because the rate of leaf appearance is a temperature-driven process (Hammer *et al.*, 2010), increased TLN would increase the amount of thermal units required to reach anthesis, and could also increase leaf area production if there is no effect on tillering (van Oosterom *et al.*, 2011). This may increase pre-anthesis water use, thus adversely affecting post-anthesis water availability and hence grain yield in environments with end-of-season drought stress (Borrell *et al.*, 2014; George-Jaeggli *et al.*, 2017). If changes in temperature affect the synchrony of leaf production and life cycle development differently among genotypes, this could have important implications for adaptation to a warming climate.

A previous study (Tirfessa *et al.*, 2020) identified significant genotypic differences in the response of phenology (pre-anthesis DR) to temperature amongst diverse Ethiopian germplasm. Although Parent *et al.* (2019) warn that such differences could be associated with confounding effects in the analyses, the observation that these differences were associated with differences in the base temperature (*T*_base_) that were linked to racial grouping adds veracity to these results. In addition, significant genotypic differences in LAR and in the effect of temperature on TLN have been reported for sorghum genotypes that included germplasm of Ethiopian background (van Oosterom *et al.*, 2011). However, no studies have been conducted that link genotypic differences in the response of TLN to temperature to underpinning genotypic differences in the responses of LAR and DR to temperature. Hence, the objectives of this study were to use a set of diverse Ethiopian sorghum germplasm to (1) establish the presence of temperature effects on TLN, (2) develop a crop physiological framework that could explain this by determining differences in temperature responses between LAR and pre-anthesis DR, and (3) discuss implications for specific adaptation to agroecological zones and hence breeding.

## MATERIALS AND METHODS

### Experiment details

**Fig. 1.**
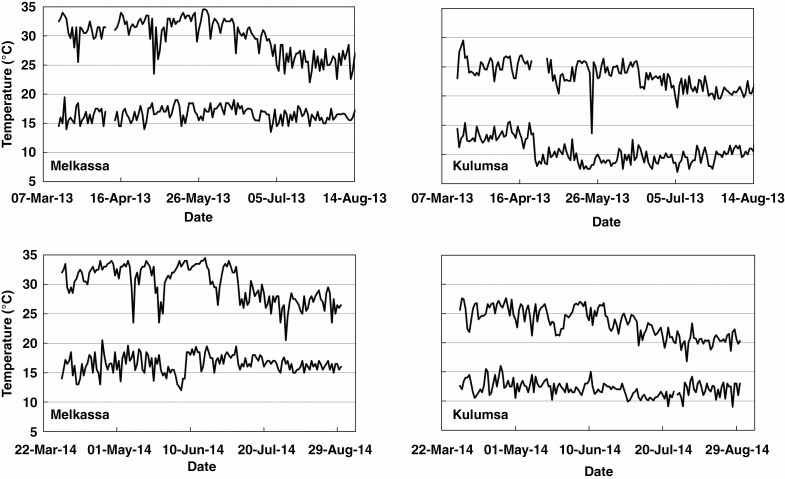
Daily maximum and minimum temperatures at Melkassa and Kulumsa for the 2013 and 2014 seasons.

Experiments were conducted at two locations in Ethiopia with comparable latitude but contrasting altitude: Melkassa (1046 m, 8°25ʹN, 39°19ʹE) and Kulumsa (2259 m, 8°01ʹN, 39°09ʹE), which represent lowland and highland altitudes respectively. The sorghum [*Sorghum bicolor* (L.) Moench] genotypes were sown on 12 different dates in 2013 and 2014, with six sowing dates per year per location. Sowings were conducted at ~3- week intervals, ranging from 24 March to 8 July in 2013 and 12 April to 25 July in 2014. The difference in sowing dates between locations was generally no more than 2 d, with only one occurrence of a 4-d difference (Table 1). Combined with the difference in altitude between locations, this created differences in temperatures that were independent of photoperiod. Daily temperatures at Melkassa ranged from ~15 to 34 °C and at Kulumsa from ~10 to 25 °C (Fig. 1). During the 4 weeks after sowing, which covers most of the period prior to PI, when TLN is determined (van Oosterom *et al.*, 2010), average temperatures at Melkassa were 4.4–8.3 °C higher than at Kulumsa (Table 1).

**Table 1.** Date of sowing and average daily maximum (*T*_max_) and minimum (*T*_min_) temperature during the 4 weeks^1^ following sowing for each of the 12 sowing dates at Melkassa (low altitude) and Kulumsa (high altitude). The last column gives the average difference in daily mean temperature between the two locations, with positive values indicating higher mean daily temperature at Melkassa.

	Melkassa			Kulumsa		Difference in mean temp. (°C)
Sowing date	*T*_max_ (°C)	*T*_min_ (°C)	Sowing date	*T*_max_ (°C)	*T*_min_ (°C)	
24-Mar-13	31.1	16.2	24-Mar-13	25.2	13.4	4.37
15-Apr-13	31.4	16.6	15-Apr-13	24.3	10.8	6.49
07-May-13	32.6	17.1	07-May-13	24.1	9.0	8.29
27-May-13	31.6	17.4	27-May-13	24.2	9.3	7.75
17-Jun-13	28.2	16.1	17-Jun-13	22.1	9.0	6.62
08-Jul-13	25.6	16.2	08-Jul-13	21.1	9.7	5.48
12-Apr-14	32.2	16.7	11-Apr-14	25.7	12.9	5.15
03-May-14	30.9	16.7	02-May-14	24.3	12.5	5.35
23-May-14	32.0	16.3	23-May-14	25.0	12.2	5.52
14-Jun-14	30.9	17.3	16-Jun-14	23.3	11.5	6.67
04-Jul-14	27.5	16.8	08-Jul-14	21.1	10.7	6.24
25-Jul-14	26.6	16.1	25-Jul-14	20.3	11.6	5.45

^1^This approximates the period from emergence to panicle initiation, during which total leaf number is determined.

Each experiment (location × sowing date) was set up as a randomized complete block design with two replications, with a different randomization for each experiment. Plots consisted of a single row of 5 m length with a row spacing of 0.75 m and 1.5 m distance between blocks. Seeds were manually drilled into the rows and seedlings were thinned to 0.15 m between plants at ~20 d after emergence. Phosphorus fertilizer (46 kg ha^−1^ P_2_O_5_) in the form of di-ammonium phosphate and nitrogen fertilizer (23 kg ha^−1^ nitrogen as urea) were applied at sowing and at 35 d after sowing, respectively. All experiments were well watered.

### Genetic material

A set of 19 diverse Ethiopian genotypes that included landraces and improved varieties was evaluated in this study (Table 2). The genotypes represented three major sorghum races and included seven Ethiopian highland *durra* types, five *caudatum* types and four *kafir* types (Table 2). For 12 of these 16 genotypes, at least 99 % of their genome belonged to a single racial group. The four exceptions were Macia (96.9 % *caudatum*), Birmash (94.1 % *kafir*), Jamiyu (91.2 % Ethiopian highland *durra*) and Geremew (80.7 % *kafir*). The last also contained 11.0 % Ethiopian highland *durra* and 7.1 % *caudatum* (Tirfessa *et al.*, 2020). In addition, two of the genotypes were *caudatum*/*guinea* mixed race: Adukura (50 % *caudatum* and 22 % *guinea*) and Bobe red (63 % *caudatum* and 22 % *guinea*). For ESH-2, the racial classification was not available. Racial classification was closely associated with the four major sorghum agroecological growing areas in Ethiopia. These areas differ in altitude (metres above sea level, m asl), rainfall (mm) and duration of the growing period (d), and comprise the highlands (>1900 m asl, 800 mm, 170–200 d), intermediate zone (1600–1900 m asl, >1000 mm, 150–180 d), wet lowlands (<1600 m asl, >1000 mm, 110–150 d) and dry lowlands (<1600 m asl, <600 mm, 90–130 d) (Ayana and Bekele, 2000). The four highland genotypes were all *durra*, the four genotypes grown at intermediate altitude were all *kafir*, the two genotypes from the wet lowlands were both *caudatum/guinea*, whereas the dry lowland genotypes were either *durra* or *caudatum* (Table 2).

**Table 2.** Mean, minimum and maximum values for total leaf number at Melkassa and Kulumsa plus the average difference between the two locations across 12 sowing dates for the 19 Ethiopian (ETH) genotypes. Significance relates to the difference in average leaf number between the two sites. Within each of the two response patterns, genotypes have been grouped by race and germplasm type.

				Total leaf number		
Genotype	Race	Germplasm type	Agroecology	Melkassa	Kulumsa	Difference^2^
				Mean (range)	Mean (range)	
**Genotypes with significantly greater leaf number at Melkassa than at Kulumsa**						
Chiro	ETH highland *durra*	Improved landrace	Highland	27.1 (24.8–30.7)	23.3 (21.7–26.5)	3.76***
Chelenko	ETH highland *durra*	Improved landrace	Highland	26.7 (23.3–29.8)	23.4 (21.3–25.5)	3.31***
ETS2752	ETH highland *durra*	Improved landrace	Highland	26.0 (23.2–31.0)	23.0 (20.3–26.0)	3.05***
Alemaya70	ETH highland *durra*	Improved landrace	Highland	24.5 (21.7–28.2)	22.5 (19.3–25.8)	1.99**
Jamiyu	ETH highland *durra*	Landrace	Dry lowland	21.6 (19.2–24.3)	18.8 (17.2–21.2)	2.81***
Degalit	ETH highland *durra*	Landrace	Dry lowland	23.4 (20.8–27.0)	21.8 (20.3–23.8)	1.68*
ESH2	NA^1^	Released	Dry lowland	15.2 (12.3–17.0)	13.2 (11.3–14.3)	2.29***
Adukara	*caudatum*/*guinea*	Landrace	Wet lowland	26.7 (22.1–31.7)	25.2 (19.3–29.2)	1.58**
Gambella1107	*caudatum*	Released	Dry lowland	18.0 (15.8–20.2)	16.2 (15.0–17.8)	1.88***
Melkam	*caudatum*	Released	Dry lowland	16.3 (14.0–18.5)	14.8 (12.7–19.2)	1.55*
Teshale	*caudatum*	Released	Dry lowland	15.8 (13.5–17.5)	14.3 (13.0–15.8)	1.47**
**Genotypes with leaf number at Melkassa not greater than at Kulumsa**						
Jigurti	ETH highland *durra*	Landrace	Dry lowland	20.0 (17.3–22.5)	19.0 (17.7–20.7)	0.99 n.s.
Bobe red	*caudatum*/*guinea*	Landrace	Wet lowland	24.9 (19.5–29.9)	23.9 (18.5–26.3)	1.07 n.s.
Macia	*caudatum*	Released	Dry lowland	16.2 (14.0–18.2)	15.6 (14.3–17.8)	0.61 n.s.
Meko	*caudatum*	Released	Dry lowland	15.0 (12.7–17.0)	15.1 (13.0–19.5)	−0.10 n.s.
Geremew	*kafir*	Released	Intermediate	20.9 (18.7–23.3)	19.8 (17.7–21.7)	1.06 n.s.
Birmash	*kafir*	Released	Intermediate	19.7 (17.7–22.3)	19.8 (17.5–21.0)	−0.16 n.s.
IS9302	*kafir*	Released	Intermediate	19.3 (16.0–21.8)	19.7 (18.0–21.3)	−0.36 n.s.
Dagem	*kafir*	Released	Intermediate	20.5 (16.8–24.2)	21.4 (18.7–23.8)	−0.98*
Race means^2^	Race	No. of genotypes				
	ETH highland *durra*	7				2.51 a
	*caudatum*/*guinea*	2				1.33 ab
	*caudatum*	5				1.08 b
	*kafir*	4				-0.11 b

^1^NA, not available.

^2^Racial means followed by a different letter differ significantly (*P* < 0.05) according to a *t*-test using the pooled method for equal variances.

Asterisks indicate significance at *P < 0.05, **P < 0.01 and ***P < 0.001. n.s., not significant.

### Observations

In each experiment, observations were conducted on three plants per plot that were tagged when about five leaves had fully expanded. Main shoot TLN was recorded in all experiments once the flag leaf was fully expanded. In addition, the number of fully expanded main shoot leaves throughout the vegetative period was counted at regular intervals in all experiments in 2014. A leaf was considered as fully expanded when its ligule became visible above the enclosing sheath of the previous leaf (Hammer *et al.*, 1993). Leaf counts were done up to twice a week in the first sowing dates, but at lower frequencies in later sowing dates. Weather data (daily maximum and minimum temperature) were collected from a weather station that was located in close proximity to the experiment.

### Data analysis

#### Total leaf number (TLN).

An analysis of variance (ANOVA) for TLN was conducted to determine if there were any effects of location and genotype on TLN across pairs of experiments with common sowing dates. Hence, the 24 experiments (2 locations × 12 sowing dates across two seasons) were analysed in terms of sowing date (11 d.f.) and location within sowing date (12 d.f.). The presence of significant genotype and location effects on TLN was subsequently analysed in more detail using a paired-sample *t*-test for TLN across all 12 sowing dates for each individual genotype, to identify genotypes with a significant location effect on TLN. The TLN data were analysed using the ANOVA and TTEST procedures in SAS Enterprise Guide 9.4 (SAS, 2013).

#### Leaf appearance rate (LAR).

For each genotype in each of the 12 experiments in 2014, the average number of fully expanded leaves was plotted against the number of days after sowing (DAS). Leaf number was the average value across the two replications for each experiment (six plants per genotype). For the period during which leaf number was linearly related to DAS, the slope of the regression was taken as the LAR (leaves d^−1^). Because temperatures were relatively constant for large periods of time during the growing season (Fig. 1), a single regression generally sufficed for most of the period of leaf appearance (see Results). Observations close to maximum TLN were excluded from the regression, as these leaf counts could potentially have been biased by some of the plants having reached flag leaf. The first observation(s) were also excluded from the regression in cases where these early leaf counts had a disproportionate effect on the slope of the regression (LAR, leaves d^−1^). For some early-flowering genotypes, the number of observations was too limited in some experiments to reliably determine LAR. For situations with LAR data, weather data were then used to calculate the average daily temperature for the period during which leaf number was linearly related to DAS. The data for LAR and mean daily temperature that were thus obtained for each genotype in each experiment were then plotted against each other in order to determine the response of LAR (leaves d^−1^) to mean daily temperature. Each regression had up to 12 data points (2 locations × 6 sowing dates). The intercept of the regression with the *x*-axis was considered to be the base temperature (*T*_base_) for LAR and represented the average daily temperature at which LAR is zero. The slope of the regression (leaves d^−1^ °C^−1^) represents the increase in LAR (leaves d^−1^) for each degree increase in average daily temperature. To determine if genotypes differed in *T*_base_, the slope of the regression or both, the following fixed effect non-linear model was fitted:


$$\begin{array}{l} LAR_{ijk}=\;b\left(T_{ijk}\;T_{base}\right)\;+\varepsilon_{ijk} \end{array}$$ (1)

where *b* is the slope of the relationship, *T*_base_ = α/β, with α and β the intercept and slope respectively for the simple linear expression of LAR on *T*_av_, and ɛ_*ijk*_ ~ N(0, σ^2^). Analyses were done in R version 4.05 (R Core Team, 2021) using the non-linear least squares (*nls*) function (Ritz and Streibig, 2008) to estimate the parameters. Equation (1) was fitted with and without genotype-specific values for *T*_base_ and the slope of the temperature response, and the significance of genotype-specific parameter values was determined through model comparisons.

The *T*_base_ for LAR was also compared with the *T*_base_ for pre-anthesis DR (Tirfessa *et al.*, 2020) to determine if differences between the two processes for individual genotypes could account for genotypic differences in the response of TLN to location (Melkassa vs. Kulumsa). A higher *T*_base_ and responsiveness to temperature for LAR than for DR could indicate that an increase in temperature would have a relatively larger effect on LAR than on rate of development. As LAR is associated with LIR (Padilla and Otegui, 2005), this could result in more leaves being initiated at higher temperatures, resulting in greater TLN. All regressions were conducted using the REG and NLIN procedures in SAS Enterprise Guide 9.4 (SAS, 2013).

## RESULTS

### Location effect on TLN in some genotypes

**Fig. 2.**
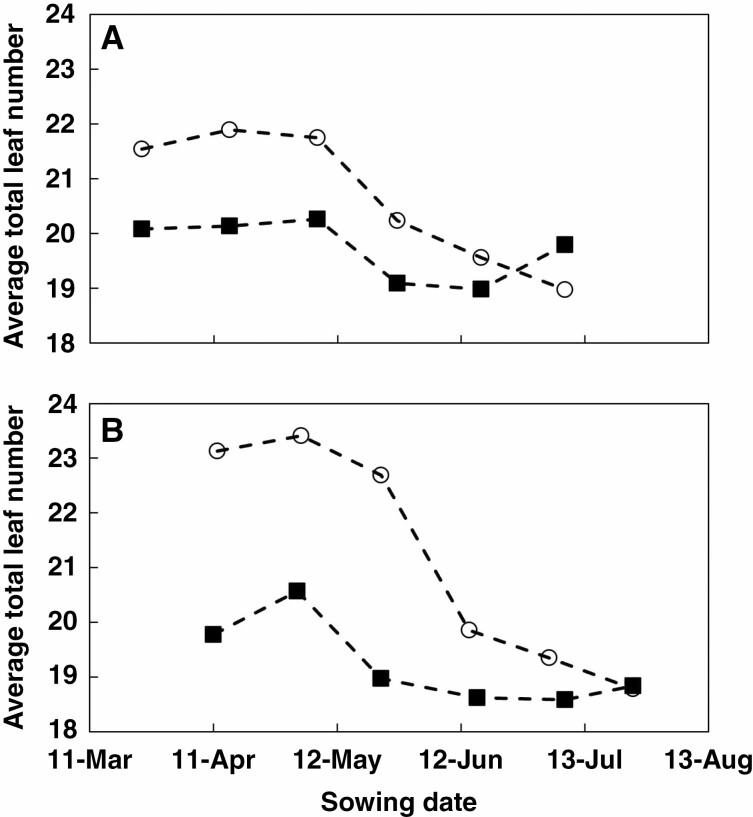
Average total leaf number (TLN) vs. sowing date at Melkassa (○) and Kulumsa (■) in (A) 2013 and (B) 2014.

Genotypic differences in TLN were highly significant (Table 3). Average TLN at Melkassa ranged from 15.0 (Meko) to 27.1 (Chiro) and at Kulumsa from 13.2 (ESH2) to 25.2 (Adukara) (Table 2). In general, TLN at Melkassa exceeded TLN at Kulumsa, particularly for the first three sowing dates in each year (Fig. 2). The sowing date and location within sowing date each captured around half of the total sum of squares across the 24 experiments and both effects were highly significant (*P* < 0.0001; Table 3). Genotypes differed in their response of TLN to location (daily mean temperature), as indicated by the highly significant (*P* < 0.0001) genotype × experiment interaction and in particular the highly significant (*P* < 0.0001) genotype × location (sowing date) interaction (Table 3).

**Table 3.** Analysis of variance for total leaf number for 19 genotypes, grown in 24 experiments that comprised 12 sowing dates (sow) × two locations (loc), with near-similar sowing dates across locations. The experimental effect was analysed in terms of sowing date and location within sowing date. Each experiment (location × sowing date) was set up as a randomized complete block design with two replications, with a different randomization for each experiment

Source	d.f.	Sum of squares	*F* value	*P* value
genotype	18	13 026	683.15	<0.0001
exp	23	1771	72.68	<0.0001
sow	11	952	81.72	<0.0001
loc(sow)	12	819	68.22	<0.0001
geno × exp	410	1663	3.83	<0.0001
geno × sow	198	992	4.73	<0.0001
geno × loc(sow)	212	671	3.17	<0.0001
rep(experiment)	24	31	1.23	0.2119

To explore the genotypic differences in the response of TLN to location in more detail, a pairwise *t*-test across the 12 sowing dates was conducted for each genotype. The results showed that 11 genotypes had significantly greater TLN at Melkassa than at Kulumsa (Table 2). Among the remaining eight genotypes, seven had no significant location effect, whereas Dagem had significantly lower TLN at Melkassa than at Kulumsa. The response of TLN to location (temperature) had some association with racial background. Six of the seven highland *durra* genotypes produced significantly greater TLN in the warmer conditions of Melkassa compared to the cooler conditions of Kulumsa, with three of these (Chiro, Chelenko and ETS2752) on average producing >3 more leaves at Melkassa than at Kulumsa (Table 2). In contrast, none of the four *kafir* genotypes showed such a response. The difference in the location effect on TLN between the *durra* and *kafir* groups was highly significant (*P* < 0.01) according to a *t*-test. However, the seven *caudatum* and *caudatum*/*guinea* genotypes were more evenly distributed between the two groups (Table 2).

### Response of LAR to temperature

**Fig. 3.**
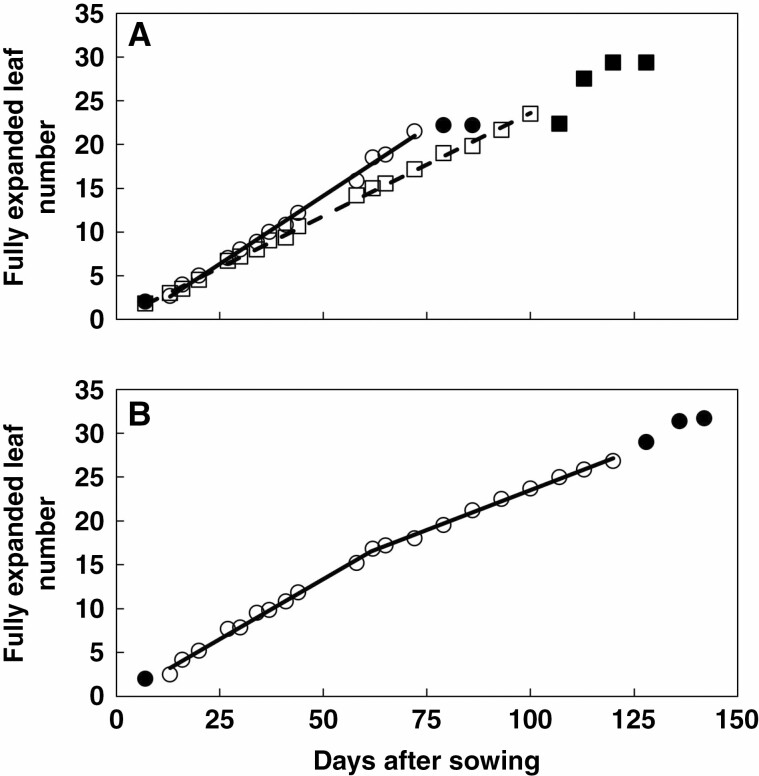
Fully expanded leaf number (FEL) vs. days after sowing (DAS) for sowing 1 at Melkassa in 2014 for (A) Jigurte (○—○) and Bobe red (□- - - □) and (B) Adukara (○—○). Data are averaged across two replications (six plants). Closed symbols have been excluded from the regressions. Equations:

The relationship between fully expanded leaf number and days after sowing was generally linear until close to full flag leaf appearance for most of the genotype × experiment combinations. This is illustrated in Fig. 3A for Jigurte and Bobe red, sown at Melkassa on 12 April, 2014. The only exception was Adukara, for which a bilinear relationship generally gave a significantly better fit than a linear relationship (Fig. 3B). Because Adukara was one of the genotypes that produced most leaves (Table 2) and was amongst the latest to reach anthesis (Tirfessa *et al.*, 2020), it is possible that this bilinear relationship reflected a delayed onset of stem elongation, where the first slope represented the period when the growing point was still below the soil surface. Hence, to facilitate comparisons with other genotypes, the second slope was used for further analyses. Across all genotype × experiment combinations, the average number of observations in each regression used to derive LAR was 9.08 (range 3–23) and the average *R*^2^ was 0.995 (range 0.965–1.000). Across all experiments, the average daily temperature ranged from ~16.0 to 24.1 °C, although the range was slightly lower for Adukara (16.0–22.9 °C), where only data for the second slope (Fig. 3B) were used.

An ANOVA showed that for the relationship between LAR and daily average temperature, genotypes differed significantly for both slope and *T*_base_. A model that assumed a genotype-specific slope but common *T*_base_ across genotypes (eqn 1) gave a significantly (*P* < 0.0001) better fit than a common regression across all genotypes. Similar results were obtained with a model that assumed a genotype-specific *T*_base_ but a common slope across genotypes. Importantly, however, in both cases, the model improved significantly (*P* < 0.05) if genotype-specific values for both slope and *T*_base_ were used. Moreover, a common *T*_base_ of 11 °C, commonly used as the *T*_base_ of phenological processes for Australian sorghum (Hammer *et al.*, 1993), gave a fit that was significantly worse (*P* < 0.0001) than that of using genotype-specific values. This indicates that it was appropriate to fit separate slopes and *T*_base_ in eqn (1) for each genotype (Table 4).

**Table 4.** Statistics for the regression of leaf appearance rate (LAR, leaves d^−1^) on average daily temperature for key Ethiopian (ETH) sorghum germplasm. Statistics include the number of experiments (*n*, sowing date × location) for which LAR could be estimated reliably, goodness of fit (*R*^2^), slope of the response (leaves d^−1^ °C^−1^) ± s.e., estimated base temperature (*T*_base_) ± s.e. for LAR, and estimated LAR at 24 °C. The final two columns indicate estimated *T*_base_ for pre-anthesis development rate (DR) (Tirfessa *et al.*, 2020) and the difference in *T*_base_ between LAR and DR. Genotypes have been classified according to whether leaf number at Melkassa was significantly greater than at Kulumsa (Table 2).

Genotype	Race	*n* ^1^	*R* ^2^	Slope (leaves d^−1^ °C^−1^)	*T*_base_ LAR (°C)	LAR at 24 °C (leaves d^−1^)	*T*_base_ DR (°C)	*T*_base_ difference (°C)
**Genotypes with significantly greater leaf number at Melkassa than at Kulumsa**								
Chiro	ETH highland *durra*	12	0.87	0.0143 ± 0.0018	4.0 ± 2.0	0.286	1.5	2.5
Chelenko	ETH highland *durra*	12	0.83	0.0145 ± 0.0018	4.0 ± 1.9	0.290	0.1	3.9
ETS2752	ETH highland *durra*	12	0.86	0.0146 ± 0.0017	4.1 ± 1.9	0.292	0.0	4.1
Alemaya 70	ETH highland *durra*	12	0.91	0.0145 ± 0.0017	3.9 ± 1.9	0.293	3.4	0.5
Jamiyu	ETH highland *durra*	12	0.82	0.0164 ± 0.0017	5.8 ± 1.5	0.300	5.4	0.4
Degalit	ETH highland *durra*	11	0.91	0.0174 ± 0.0018	6.1 ± 1.4	0.311	5.5	0.6
ESH2	NA^2^	11	0.88	0.0140 ± 0.0017	3.7 ± 2.0	0.284	0.4	3.3
Adukara	*caudatum*/*guinea*	12	0.84	0.0144 ± 0.0019	9.1 ± 1.3	0.214	8.9	0.2
Gambella 1107	*caudatum*	11	0.84	0.0147 ± 0.0018	4.0 ± 2.0	0.294	0.9	3.1
Melkam	*caudatum*	12	0.91	0.0114 ± 0.0017	1.8 ± 2.7	0.252	0.0	1.8
Teshale	*caudatum*	11	0.97	0.0155 ± 0.0017	5.5 ± 1.6	0.286	1.3	4.2
**Average** ^3^				0.0147*	4.72*	**0.282 n.s.**	2.49***	2.23***
**Genotypes with leaf number at Melkassa not greater than at Kulumsa**								
Jigurti	ETH highland *durra*	12	0.85	0.0172 ± 0.0016	5.7 ± 1.4	0.315	6.6	−0.9
Bobe red	*caudatum*/*guinea*	10	0.93	0.0154 ± 0.0018	6.9 ± 1.5	0.263	7.3	−0.4
Macia	*caudatum*	11	0.91	0.0155 ± 0.0017	5.3 ± 1.6	0.291	4.1	1.2
Meko	*caudatum*	11	0.91	0.0158 ± 0.0017	5.2 ± 1.6	0.296	6.0	−0.8
Geremew	*kafir*	12	0.95	0.0144 ± 0.0017	6.2 ± 1.6	0.256	7.2	−1.0
Birmash	*kafir*	12	0.93	0.0181 ± 0.0017	7.7 ± 1.1	0.294	9.8	−2.1
IS9302	*kafir*	12	0.96	0.0213 ± 0.0017	9.2 ± 0.8	0.315	9.4	−0.2
Dagem	*kafir*	12	0.93	0.0191 ± 0.0017	8.6 ± 1.0	0.294	9.0	−0.4
**Average**				**0.0171**	**6.86**	**0.290**	**7.43**	−**0.56**
Race means^4^								
	ETH highland *durra*	7		0.0156 a	4.79 b	0.298 a	3.21 b	1.57 a
	*caudatum*	5		0.0146 a	4.36 b	0.284 ab	2.46 b	1.90 a
	*caudatum*/*guinea*	2		0.0149 a	8.01 a	0.238 b	8.10 a	−0.09 ab
	*kafir*	4		0.0182 a	7.93 a	0.290 ab	8.85 a	−0.92 b

^1^Leaf counts to estimate LAR were only conducted in 2014, giving a maximum of 12 data points (6 sowing dates × 2 locations). For some of the early genotypes, one or two experiments had insufficient data points to reliably estimate LAR.

^2^NA, not available.

^3^Asterisks indicate significance at *P < 0.05 and ***P < 0.001 in the comparison of the average values for the two groups of genotypes. n.s., not significant.

^4^Means followed by a different letter differ significantly (*P* < 0.05) according to a *t*-test using the pooled method for equal variances.

**Fig. 4.**
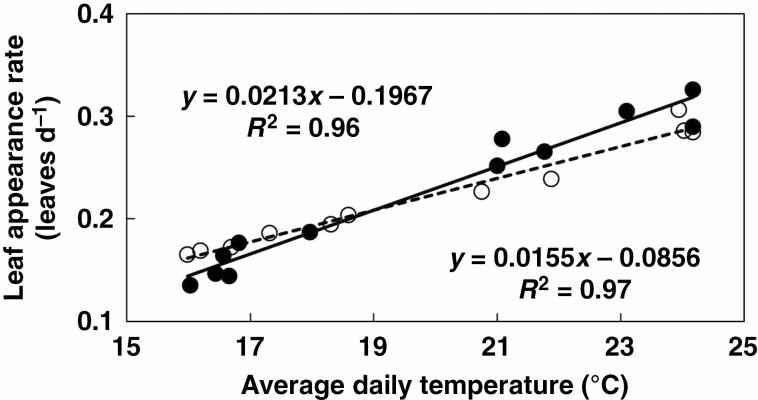
Leaf appearance rate (LAR, leaves d^−1^) vs. average daily temperature (°C) for Teshale (○- - - ○) and IS9302 (●—●). Each data point in this figure represents the LAR of one experiment, as determined from data illustrated in Fig. 3.

The response of LAR (leaves d^−1^) to average daily temperature was strongly linear across experiments (Fig. 4), with *R*^2^ ranging from 0.82–0.83 for Jamiyu and Chelenko to 0.96–0.97 for IS9302 and Teshale respectively (Table 4). The estimated *T*_base_ for LAR ranged from 1.8 °C for Melkam to 9.2 °C for IS9302 (Table 4). The *kafir* and *caudatum*/*guinea* genotypes consistently had an estimated *T*_base_ ≥ 6.2 °C, whereas the *caudatum* and highland *durra* genotypes all had an estimated *T*_base_ ≤ 6.1 °C (Table 4). As a consequence, the average *T*_base_ of the *caudatum* and highland *durra* races was significantly lower than that of the *kafir* and *caudatum*/*guinea* races. The slope of the relationship between LAR and daily average temperature ranged from 0.0114 leaves d^−1^ °C^−1^ for Melkam to 0.0213 leaves d^−1^ °C^−1^ for IS9302 (Table 4). With the exclusion of Adukara, *T*_base_ and slope were highly positively correlated (*R*^2^ = 0.83, *n* = 18), such that genotypes with high *T*_base_ increased the LAR quickest with increasing temperature. Hence, the three genotypes with the greatest slope all belonged to the *kafir* race with high *T*_base_. Nonetheless, there were no significant differences in the average slope among racial groups (Table 4). The estimated LAR at 24 °C ranged from 0.214 leaves d^−1^ (Adukara) to 0.315 leaves d^−1^ (Jigurti, IS9302). The two *caudatum*/*guinea* genotypes both had low LAR at this temperature and the average of this group was significantly lower than that of the highland *durra* genotypes (Table 4).

### Difference in base temperature between LAR and DR is linked to a location effect on TLN

**Fig. 5.**
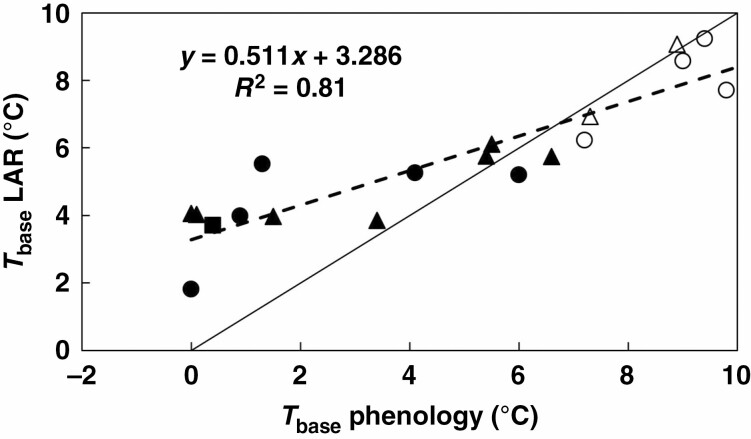
Base temperature (*T*_base_, °C) for leaf appearance rate (LAR) vs. base temperature for pre-anthesis development rate (DR) for the 19 genotypes representing the *caudatum* (●), highland *durra* (▲), *kafir* (○) and *caudatum*/*guinea* (Δ) races. ESH-2, for which the race was unknown, is represented by ■. The solid line is the 1:1 line.

**Fig. 6.**
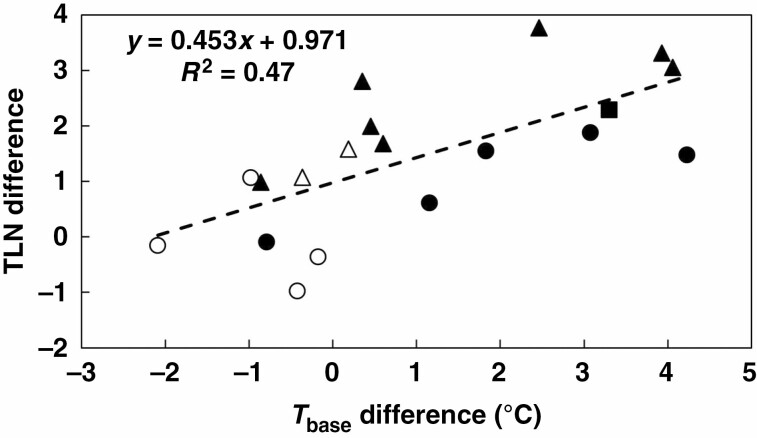
Association between average difference in total leaf number (TLN) at Melkassa and Kulumsa across 12 sowing dates vs. differences in base temperature (*T*_base_, °C) between leaf appearance rate and pre-anthesis development rate for the 19 genotypes representing the *caudatum* (●), highland *durra* (▲), *kafir* (○) and *caudatum*/*guinea* (Δ) races. ESH-2, for which the race was unknown, is represented by ■.

The veracity of the genotypic differences in *T*_base_ for LAR was supported by the observation that across all genotypes, *T*_base_ for LAR was significantly positively associated with the *T*_base_ for pre-anthesis DR (*R*^2^ = 0.81, *n* = 19, *P* < 0.0001, Fig. 5). The 11 genotypes with a significant temperature (location) effect on TLN on average had significantly lower *T*_base_ for both LAR (*P* < 0.05) and DR (*P* < 0.001) than the eight with no temperature effect on TLN (Table 4). Importantly, however, for the former group of 11 genotypes, *T*_base_ for LAR was on average 2.2 °C higher than *T*_base_ for DR (Table 4) and this value was significantly (*P* < 0.05) greater than zero based on a *t*-test for pairwise comparisons. In contrast, for the other eight genotypes *T*_base_ for LAR was, with the exception of Macia (Table 4), slightly lower than *T*_base_ for DR. Importantly, the average difference of 0.6 °C was not significantly different from zero. These differences in *T*_base_ between LAR and DR were partly associated with racial grouping. The four *kafir* genotypes, which had significantly higher *T*_base_ for both LAR and DR than the highland *durra* and *caudatum* genotypes, also had a significantly smaller difference in *T*_base_ between these two processes than the *durra* and *caudatum* groups (Table 4). Across all 19 genotypes, the difference in TLN between Melkassa and Kulumsa was positively associated with the difference in *T*_base_ between LAR and DR (*R*^2^ = 0.47, *n* = 19, *P* < 0.01, Fig. 6). These results thus indicate that genotypes with a significant response of TLN to temperature (as indicated by the location effect on TLN) had a greater difference in *T*_base_ between LAR and DR than genotypes with no difference in TLN between Melkassa and Kulumsa.

## DISCUSSION

### Genotypes differed in base temperature for response of leaf appearance rate to temperature

Genotypes differed significantly in the response of LAR to temperature. Genotypic differences in LAR have been reported previously in C_4_ cereals, including maize (Padilla and Otegui, 2005) and sorghum (Major *et al.*, 1990; van Oosterom *et al.*, 2011). For sorghum, Major *et al.* (1990) found that genotypic differences in LAR were associated with interactions among the sorghum maturity genes (*Ma*_*1*_, *Ma*_*2*_, *Ma*_*3*_), with *Ma*_*3*_ having greater phyllochron (lower LAR) than *ma*_*3*_ if *ma*_*2*_ is recessive, but not if *Ma*_*2*_ is dominant, whereas *Ma*_*1*_ had greater phyllochron than *ma*_*1*_ if *Ma*_*2*_ is dominant, but not if *ma*_*2*_ is recessive. For maize, Padilla and Otegui (2005) related genotypic differences in LAR to differences in *T*_base_, but no such information is available for sorghum. The current analyses indicated that genotypic differences in the response of LAR to temperature were associated with genotypic differences in both *T*_base_ and the slope of the response, even though the two factors were highly correlated. The analyses also indicated that the *T*_base_ of 11 °C that has been derived in Australia (Hammer *et al.*, 1993) was significantly greater than values obtained in the current analyses. In addition, there were significant differences amongst racial groups in the *T*_base_ for LAR (Table 4). Moreover, across the 19 genotypes, the *T*_base_ for LAR was significantly correlated with the *T*_base_ for pre-anthesis DR (Fig. 5), which was determined concomitantly (Tirfessa *et al.*, 2020). This high correlation between the *T*_base_ of these two processes would support the view of Parent and Tardieu (2012) that temperature responses of different processes may have shifted synchronously throughout evolution. Hence, the current analyses support the hypothesis that the significant genotypic differences in the response of LAR to temperature were at least partly associated with genotypic differences in the *T*_base_ for LAR.

The racial differences in *T*_base_ for LAR were very distinct, with *caudatum* and highland *durra* genotypes consistently having lower *T*_base_ than the *kafir* and *caudatum*/*guinea* genotypes, resulting in highly significant differences in average *T*_base_ across these racial groups (Table 4). The low *T*_base_ of *durra* genotypes would explain the significantly greater LAR of hybrids based on the Ethiopian *durra* inbred line ATx642 compared to AQL39 hybrids when LAR is expressed in leaves d^−1^ (van Oosterom *et al.*, 2011). The low *T*_base_ of these genotypes allows LAR to proceed at higher rates under low temperatures. Because temperatures tend to decline with altitude (Fig. 1) this provides specific adaptation to the agroecological zone of adaptation of these highland *durra* genotypes. Conversely, the high *T*_base_ of the two *caudatum*/*guinea* genotypes (Table 4) potentially provides adaption to the agroecology of the wet lowlands (Table 2), where, in the absence of drought, biomass accumulation is likely to be radiation-limited rather than water-limited. A high *T*_base_ for LAR could potentially slow LAR, which would in turn extend the time to full flag leaf appearance and hence increase radiation interception to increase biomass accumulation and hence grain yield. This consistency between racial differences in *T*_base_ for LAR and their agroecological adaptation lends further veracity to the observed differences in *T*_base_ for LAR.

### Temperature effects on leaf number were related to differences in base temperature between leaf appearance and development

Significantly greater TLN under the high temperatures at Melkassa compared to lower temperatures at Kulumsa for similar sowing dates indicated a temperature effect on TLN that was independent of photoperiod. Similar increases in TLN under higher temperatures during the period following emergence have been observed previously in maize (Cooper and Law, 1978) and sorghum (Quinby *et al.*, 1973; Gerik and Miller, 1984; van Oosterom *et al.*, 2011). Initiation of new leaf primordia in the meristem ceases at PI, the timing of which depends on photoperiod and temperature (Hammer *et al.*, 2010). The number of leaves initiated depends on the duration of the period until PI and on LIR. If daily temperature increases, the accelerated DR towards PI will be offset by an accelerated LIR, such that if the two processes have similar *T*_base_, then TLN will be independent of daily temperature. However, if *T*_base_ for LIR exceeds *T*_base_ for DR and if the slope of the response to temperature for the two processes is comparable (e.g. both are linear) for a particular genotype, then an increase in average daily temperature will result in a relatively larger change in thermal units (°Cd) for LIR compared with DR. This will increase LIR to a relatively larger extent than DR, which in turn will increase the number of leaves that are initiated at the apical meristem prior to PI, resulting in greater TLN.

Although the current study estimated *T*_base_ for LAR, rather than for LIR, genotypic differences in the response of LAR and LIR to temperature are highly coordinated prior to PI, as observed for maize (Padilla and Otegui, 2005). Similarly, in sorghum, genotypic differences in LAR were reflected by differences in LIR (van Oosterom *et al.*, 2011). In rice, the *pla1* gene that accelerates LIR was phenotypically identified through accelerated LAR (Miyoshi *et al.*, 2004). Hence, there is ample evidence across cereals that differences in the temperature response of LAR do represent differences in the response of LIR, which is a biological necessity to ensure plants can respond in a coordinated manner to environmental cues (Parent *et al.*, 2019).

The current results (Table 4) showed significant differences in *T*_base_ across phenological processes, as the average difference in *T*_base_ between LAR and DR of 2.2 °C for the 11 genotypes with a significant temperature effect on TLN was significantly greater than zero. This implies that at low temperatures, development will progress more rapidly than leaf appearance and leaf initiation. This would reduce the number of leaf primordia initiated prior to PI, which would explain the significantly lower TLN of these genotypes at Kulumsa compared to Melkassa (Table 2; Fig. 6). This is consistent with the theory that TLN increases with higher temperature for the period prior to PI (which is around 4 weeks from emergence; van Oosterom *et al.*, 2010) if *T*_base_ for LAR exceeds *T*_base_ for DR.

To our knowledge, this is the first report for sorghum that links significant genotypic differences in *T*_base_ across phenological processes to the emergent consequence of a temperature effect on TLN. Significantly, this response was linked to racial grouping. Compared with *kafir* genotypes, highland *durra* genotypes on average had significantly lower *T*_base_ for both LAR and DR, significantly greater difference in *T*_base_, and significantly greater response of TLN to temperature (Tables 2 and 4). This would explain the relatively strong response of TLN to temperature observed by van Oosterom *et al.* (2011) for hybrids based on ATx642 (formerly known as B35), which is derived from IS12555, a *durra* landrace from Ethiopia (Borrell *et al.*, 2000). However, the effect of differences in *T*_base_ on the response of TLN to temperature was not uniform, as *caudatum* genotypes on average had a *T*_base_ for both LAR and DR that was comparable with that of highland *durra* genotypes (Table 4), but the ensuing temperature effect on TLN was significantly less (Table 2). As a consequence, in the regression of Fig. 6, *caudatum* genotypes were consistently located on or below the regression line and highland *durra* genotypes on or above the regression line. The reason for this distinct behaviour is unclear. The consistent racial effect on *T*_base_ for LAR and DR, and on the difference in *T*_base_ between these two processes (Table 4), contradicts the observation of Parent and Tardieu (2012) that a general lack of genetic variability in temperature responses exists within species. Rather, the location (temperature) effect on TLN in this study indicates that sorghum genotypes can have different temperature responses across these two phenological processes.

### Implications for plant breeding and environmental adaptation

The analyses showed that significant genotypic differences in the response of LAR to temperature exist for sorghum, and that individual genotypes can have different temperature responses across processes, resulting in a response of TLN to the temperature environment. Although the study was conducted with Ethiopian germplasm, the association between temperature response and racial groups indicates generic associations that would facilitate incorporation of the current insights into any sorghum breeding programme through selection of targeted racial groups.

Genotypic differences in *T*_base_ for LAR and pre-anthesis DR can potentially affect adaptation to post-anthesis drought. A lower *T*_base_ for LAR would accelerate leaf appearance. Increased LAR can increase early vigour of the main shoot, and the resulting shift in carbon supply–demand balance of the plant can reduce tillering (van Oosterom *et al.*, 2011; Alam *et al.*, 2014). This can reduce canopy size, which in turn can reduce pre-anthesis water use (Borrell *et al.*, 2014; George-Jaeggli *et al.*, 2017). A lower *T*_base_ for DR could accelerate progress to anthesis, which can also reduce pre-anthesis water use (van Oosterom *et al.*, 2011). In environments where post-anthesis drought is likely, such savings in pre-anthesis water use can increase grain yield, which is highly correlated with post-anthesis water availability (Turner, 2004; Hammer, 2006; Borrell *et al.*, 2014). However, the trade-off of a reduced canopy size is that in well-watered conditions, where productivity is radiation-limited, this is likely to reduce intercepted radiation and hence productivity (Hammer *et al.*, 2010). Considered in isolation, a low *T*_base_ for either LAR or DR could thus be beneficial to adaptation to post-anthesis drought stress.

However, the observation that genotypes with low *T*_base_ values had the greatest difference in *T*_base_ between processes (Fig. 5) can complicate the consequences of low *T*_base_ on adaptation to drought, particularly under high temperatures. Because of the association between LAR and LIR (Padilla and Otegui, 2005), a low *T*_base_ for LAR will probably result in the initiation of more leaves prior to PI, although the extent of that will depend on the slope of the response of LAR to temperature. Any effect on TLN will be exacerbated under high temperatures, as the genotypes with low *T*_base_ for LAR and DR tend to have the greatest difference in *T*_base_ between these two processes and are thus most likely to increase TLN if temperatures increase (Tables 2 and 4; Fig. 6). An increase in TLN can potentially increase canopy size (if there is no associated reduction in tillering) and is likely to delay flowering if there is no effect on LAR. Both these effects would offset the water saving resulting from low *T*_base_ per se. Interestingly, there appeared to be some racial differences in this response, as *caudatum* genotypes generally had a smaller increase in TLN for a given difference in *T*_base_ between LAR and DR than highland *durra* and *caudatum*/*guinea* genotypes (Fig. 6). This could provide some genetic means to mitigate any increases in TLN and hence canopy size. As TLN is determined at PI, which in sorghum typically occurs around 4 weeks after emergence, any potential effects of increased temperature on TLN could also be mitigated by earlier sowing in spring, which would be facilitated by the lower *T*_base_. However, the consequences of such changes in agronomy on grain yield, particularly across cropping regions with variable timing and intensity of drought and heat stress, can be complex and would require targeted simulation studies to unravel any interactions (Hammer *et al.*, 2014). Hence, the multi-disciplinary approach to crop improvement advocated by Chenu *et al.* (2018), which integrates trait dissection such as described in the current study with crop growth modelling and molecular genetics to elucidate quantitative trait loci for the relevant underpinning traits, would be ideally suited to identify superior genotype × management × environment combinations that could exploit the potential benefits of specific adaptation of the current results.


$$\begin{array}{l} Jigurte:FEL=0.0754+0.2355*DAS;n\;=\;18;R^{2}\;=\;0.998 \end{array}$$

$$\begin{array}{l} Bobe\;red:FEL=1.3992+0.2243*DAS;n\;=\;20;R^{2}\;=\;0.988 \end{array}$$

$$\begin{array}{l} \begin{array}{ll} & Adukara:FEL=-0.3264+0.2743*\\ & DAS\;if\;DAS\;<\;61.5;elseFEL=5.4069+0.1811*DAS;\\ & n\;=\;20;R^{2}\;=\;0.998. \end{array} \end{array}$$

## References

[CIT0001] Alam MM , Hammer GL, van Oosterom EJ, Cruickshank AW, Hunt CH, Jordan DR. 2014. A physiological framework to explain genetic and environmental regulation of tillering in sorghum. New Phytologist 203: 155–167. 24665928 10.1111/nph.12767

[CIT0002] Ayana A , Bekele E. 2000. Geographical patterns of morphological variation in sorghum (*Sorghum bicolor* (L.) Moench) germplasm from Ethiopia and Eritrea: quantitative characters. Euphytica 115: 91–104.

[CIT0003] Borrell AK , Hammer GL, Douglas ACL. 2000. Does maintaining green leaf area in sorghum improve yield under drought? I. Leaf growth and senescence. Crop Science 40: 1026–1037. doi:10.2135/cropsci2000.4041026x.

[CIT0004] Borrell AK , Mullet JE George-Jaeggli B, et al. 2014. Drought adaptation of stay-green sorghum is associated with canopy development, leaf anatomy, root growth, and water uptake. Journal of Experimental Botany 65: 6251–6263. 25381433 10.1093/jxb/eru232 PMC4223986

[CIT0005] Caddel JL , Weibel DE. 1971. Effect of photoperiod and temperature on the development of sorghum. Agronomy Journal 63: 799–803. doi:10.2134/agronj1971.00021962006300050043x.

[CIT0006] Chenu K , van Oosterom EJ, McLean G, et al. 2018. Integrating modelling and phenotyping approaches to identify and screen complex traits – Illustration for transpiration efficiency in cereals. Journal of Experimental Botany 69: 3181–3194. doi:10.1093/jxb/ery059. 29474730

[CIT0007] Cooper PJM , Law R. 1978. Enhanced soil temperature during very early growth and its association with maize development and yield in the Highlands of Kenya. Journal of Agricultural Science, Cambridge 89: 569–577.

[CIT0008] Craufurd PQ , Qi A, Ellis RH, Summerfield RJ, Roberts EH, Mahalakshmi V. 1998. Effect of temperature on time to panicle initiation and leaf appearance in sorghum. Crop Science 38: 942–947. doi:10.2135/cropsci1998.0011183x003800040011x.

[CIT0009] Ellis RH , Qi A, Craufurd PQ, Summerfield RJ, Roberts EH. 1997. Effects of photoperiod, temperature and asynchrony between thermoperiod and photoperiod on development to panicle initiation in sorghum. Annals of Botany 79: 169–178.

[CIT0010] George-Jaeggli B , Mortlock MY, Borrell AK. 2017. Bigger is not always better: reducing leaf area helps stay-green sorghum use soil water more slowly. Environmental and Experimental Botany 138: 119–129. doi:10.1016/j.envexpbot.2017.03.002.

[CIT0011] Gerik TJ , Miller FR. 1984. Photoperiod and temperature effects on tropically and temperately-adapted sorghum. Field Crops Research 9: 29–40. doi:10.1016/0378-4290(84)90004-2.

[CIT0012] Hammer GL. 2006. Pathways to prosperity: breaking the yield barrier in sorghum. The Journal of the Australian Institute of Agricultural Science and Technology 19: 16–22.

[CIT0013] Hammer GL , Carberry PS, Muchow RC. 1993. Modelling genotypic and environmental control of leaf area dynamics in grain sorghum. I. Whole plant level. Field Crops Research 33: 293–310. doi:10.1016/0378-4290(93)90087-4.

[CIT0014] Hammer GL , McLean G, Chapman S, et al. 2014. Crop design for specific adaptation in variable dryland production environments. Crop and Pasture Science 65: 614–626. doi:10.1071/cp14088.

[CIT0015] Hammer GL , van Oosterom EJ, McLean G, et al. 2010. Adapting APSIM to model the physiology and genetics of complex adaptive traits in field crops. Journal of Experimental Botany 61: 2185–2202. doi:10.1093/jxb/erq095. 20400531

[CIT0016] Kholová J , McLean G, Vadez V, Craufurd P, Hammer GL. 2013. Drought stress characterization of post-rainy season (*rabi*) sorghum in India. Field Crops Research 141: 38–46. doi:10.1016/j.fcr.2012.10.020.

[CIT0017] Major DJ , Rood SB, Miller FR. 1990. Temperature and photoperiod effects mediated by the sorghum maturity genes. Crop Science 30: 305–310. doi:10.2135/cropsci1990.0011183x003000020012x.

[CIT0018] Messina CD , Podlich D, Dong Z, Samples M, Cooper M. 2011. Yield–trait performance landscapes: from theory to application in breeding maize for drought tolerance. Journal of Experimental Botany 62: 855–868. 21041371 10.1093/jxb/erq329

[CIT0019] Miyoshi K , Ahn BO, Kawakatsu T, et al. 2004. PLASTOCHRON1, a timekeeper of leaf initiation in rice, encodes cytochrome P450. Proceedings of the National Academy of Sciences of the United States of America 101: 875–880. 14711998 10.1073/pnas.2636936100 PMC321774

[CIT0020] Morgan PW , Guy LW, Pao C-I. 1987. Genetic regulation of development in *Sorghum bicolor* III. Asynchrony of thermoperiods with photoperiods promotes floral initiation. Plant Physiology 83: 448–450. 16665266 10.1104/pp.83.2.448 PMC1056378

[CIT0021] Muchow RC , Carberry PS. 1990. Phenology and leaf area development in a tropical grain sorghum. Field Crops Research 23: 221–237. doi:10.1016/0378-4290(90)90056-h.

[CIT0022] Ong CK , Monteith JL. 1985. Response of pearl millet to light and temperature. Field Crops Research 11: 141–160. doi:10.1016/0378-4290(85)90098-x.

[CIT0023] Padilla JM , Otegui ME. 2005. Co-ordination between leaf initiation and leaf appearance in field-grown maize (*Zea mays*): genotypic differences in response of rates to temperature. Annals of Botany 96: 997–1007. doi:10.1093/aob/mci251. 16126778 PMC4247088

[CIT0024] Parent B , Millet EJ, Tardieu F. 2019. The use of thermal time in plant studies has a sound theoretical basis provided that confounding effects are avoided. Journal of Experimental Botany 70: 2359–2370. 31091318 10.1093/jxb/ery402

[CIT0025] Parent B , Tardieu F. 2012. Temperature responses of developmental process have not been affected by breeding in different ecological areas for 17 crop species. New Phytologist 194: 760–774. doi:10.1111/j.1469-8137.2012.04086.x. 22390357

[CIT0026] Paulson IW. 1969. Embryogeny and caryopsis development of *Sorghum bicolor* (L.) Moench. Crop Science 9: 97–102. doi:10.2135/cropsci1969.0011183x000900010034x.

[CIT0027] Quinby JR. 1973. The genetic control of flowering and growth in sorghum. Advances in Agronomy 25: 125–162.

[CIT0028] R Core Team. 2021. R: a language and environment for statistical computing. Vienna: R Foundation for Statistical Computing. https://www.R-project.org/

[CIT0029] Ravi Kumar S , Hammer GL, Broad I, Harland P, McLean G. 2009. Modelling environmental effects on phenology and canopy development of diverse sorghum genotypes. Field Crops Research 111: 157–165. doi:10.1016/j.fcr.2008.11.010.

[CIT0030] Ritz C , Streibig JC. 2008 . *Nonlinear regression with R*. Berlin: Springer Science and Business Media.

[CIT0031] SAS Institute Inc. 2013. SAS Version 9.4. Cary, NC: SAS Institute Inc.

[CIT0032] Tirfessa A , McLean G, Mace E, van Oosterom E, Jordan D, Hammer G. 2020. Differences in temperature response of phenological development among diverse Ethiopian sorghum genotypes are linked to racial grouping and agro-ecological adaptation. Crop Science 60: 977–990.

[CIT0033] Turner NC. 2004. Agronomic options for improving rainfall-use efficiency of crops in dryland farming systems. Journal of Experimental Botany 55: 2413–2425. doi:10.1093/jxb/erh154. 15361527

[CIT0034] van Oosterom EJ , Borrell AK, Chapman SC, Broad IJ, Hammer GL. 2010. Functional dynamics of the nitrogen balance of sorghum. I. N demand of vegetative plant parts. Field Crops Research 115: 19–28. doi:10.1016/j.fcr.2009.09.018.

[CIT0035] van Oosterom EJ , Borrell AK, Deifel KS, Hammer GL. 2011. Does increased leaf appearance rate enhance adaptation to post-anthesis drought stress in sorghum? Crop Science 51: 2728–2740. doi:10.2135/cropsci2011.01.0031.

[CIT0036] Warrington IJ , Kanemasu ET. 1983. Corn growth response to temperature and photoperiod. III. Leaf number. Agronomy Journal 75: 762–766. doi:10.2134/agronj1983.00021962007500050010x.

